# Impact of pharmacist-led intervention for reducing drug-related problems and improving quality of life among chronic kidney disease patients: A randomized controlled trial

**DOI:** 10.1371/journal.pone.0317734

**Published:** 2025-02-03

**Authors:** Roheena Zafar, Inayat Ur Rehman, Yasar Shah, Long Chiau Ming, Khang Wen Goh, Amal K Suleiman, Tahir Mehmood Khan

**Affiliations:** 1 Department of Pharmacy, Garden Campus, Abdul Wali Khan University Mardan, Mardan, Pakistan; 2 Department of Pharmacy, North West General Hospital and Research Centre, Peshawar, Pakistan; 3 Department of Clinical Pharmacy and Practice, Faculty of Pharmacy, Universiti Malaya, Kuala Lumpur, Malaysia; 4 School of Medical and Life Sciences, Sunway University, Sunway City, Malaysia; 5 Faculty of Data Science and Information Technology, INTI International University, Nilai, Malaysia; 6 Department of Pharmacy Practice, College of Clinical Pharmacy, King Faisal University, Al Ahsa, Saudi Arabia; 7 Institute of Pharmaceutical Sciences, University of Veterinary and Animal Sciences, Lahore, Pakistan; Ministry of Health, Sri Lanka, SRI LANKA

## Abstract

**Introduction:**

Chronic kidney disease (CKD) patients suffer from different comorbid conditions and are prone toward drug-related problems (DRPs) which affect their clinical parameters as well as quality of life (QoL). This study was aimed to evaluate the impact of clinical pharmacist-led interventions on the mean number of DRPs and the mean QoL score difference per patient DRPs in CKD patients.

**Method:**

An open-labeled, randomized control trial performed from April 2023 to July 2023 in the nephrology unit of a tertiary care setting in Peshawar Pakistan. Those patients who met the inclusion criteria were randomized into two groups 1:1, i.e., control and intervention group. Clinical pharmacists identified the DRPs at baseline using Pharmaceutical Care Network Europe (PCNE) 9.1 guidelines. The QoL of patients were assessed at baseline and endpoint by using the Functional Assessment of Non-Life-Threatening Conditions (FANLTC) questionnaire.

**Results:**

A total of 100 patients were recruited having 50 in each group. The pharmacist identified a total of n = 230 DRPs in the intervention group, majority of the DRPs were attributed to inappropriate drug selection according to guidelines/formulary”; “inappropriate combinations of drugs or with herbal medications or dietary supplements”; and situations where “too many different drugs or active ingredients were prescribed”. There was 46.52% reduction in the DRPs while comparing baseline and endpoint interventions suggested by pharmacist in the intervention group. The clinical pharmacist provided interventions in order to resolve the DRPs, and 37.40% interventions were accepted and fully implemented; 31.30% of the interventions were accepted and partially implemented. The clinical pharmacist identification and proposed intervention for DRPs contributed to a statistically significant improvement in QoL, from mean ±  SD scored 58.64 ±  9.10 at the baseline to 74.48 ± 10.11 at the endpoint, with a p-value of < 0.001.

**Conclusion:**

A significant improvement in the QoL and laboratory parameters for patients with CKD following clinical pharmacist-led interventions having proposed interventions were implemented successfully from baseline to endpoint; however, a considerable number of proposed interventions were not accepted and implemented.

## Introduction

Chronic kidney disease (CKD) is defined as long-term kidney impairment lasting three months or more, or characterized by an estimated glomerular filtration rate (eGFR) less than 60 mL/min/1.73 m^2^. It affects 10–15% of the world’s population [1,2]. Despite advancements in healthcare, developing countries are witnessing a rapid increase in both the global incidence and mortality rates of CKD, with the incidence rate four times higher compared to developed countries [2]. It is estimated that the global prevalence of CKD significantly increased by 87% from 1990 to 2016, particularly among the elderly and patients with chronic diseases [3,4]. From 1990 to 2017, the global mortality rate (all-age) from CKD increased by 41.5% [4].

In Pakistan, the estimated prevalence of CKD falls within the range of 12.5–31.2% [5]. A systematic review conducted in the country estimated that CKD affects 23.3% of the Pakistani population [6]. Polypharmacy and complex drug regimens are often unavoidable in CKD patients due to several risk factors, including advanced age, female gender, and the presence of comorbidities such as hypertension, diabetes, electrolyte abnormalities, mineral bone disorders, and cardiovascular issues.

This situation leads to an increasing occurrence of drug-related problems (DRPs), such as incorrect dosages, drug-drug interactions, adverse drug reactions (ADRs), and a rising need for regular blood chemistry monitoring. These issues contribute significantly to morbidity and mortality rates and impose unnecessary financial burdens on the healthcare system [7–9]. Hypertension and diabetes mellitus are thought to be the main causes of CKD [10,11]. According to a recent study in Pakistan, hypertension and diabetes were linked to hypertensive and diabetic nephropathy in 15.2% and 27.1% of patients, respectively [12]. Older patients are at a greater risk of developing CKD; approximately 43.6% of patients over the age of 50 in Pakistan have been diagnosed with CKD [13]. However, research on the gender-specific prevalence of CKD among males and females in Pakistan is inconsistent [6].

Hospitalized CKD patients are more prone to DRPs because of their complex medical conditions, and exposure to numerous drugs. Furthermore, there have been reports indicating that the presence of multiple concomitant drugs can compromise drug adherence in CKD patients, resulting in disease progression and rehospitalization [14], and consequently a compromised QoL [15–17]. Owing to the high burden of comorbidities and medication complexity associated with CKD, effective management of CKD patients necessitates the collaboration of a multidisciplinary team of healthcare specialists, including pharmacists [18,19]. According to the existing literature, including clinical pharmacists in the multidisciplinary team has the potential to significantly increase the team’s ability to promote safe, effective, and economical drug therapy to patients [18–20]. In Pakistan, literature indicates a significant prevalence of DRPs among CKD patients. One study reported that 86.1% of CKD patients experienced DRPs, with high dosage being the most common issue (53.5%), followed by ADRs (50.5%) [21]. Additionally, other studies conducted in Pakistan found that the incidence of Potential Drug-Drug Interactions (pDDIs) among CKD patients was 84.7% [22] and 71.1% of antimicrobials were inaccurately adjusted in CKD patients [23].

In Pakistan, the implementation of comprehensive pharmaceutical care models involving pharmacists is rare, often limited to dispensing medications and providing drug counseling. Consequently, there is a need to determine the impact of clinical pharmacist services on the care of hospitalized patients with CKD. Therefore, the aim of this study was to evaluate the impact of clinical pharmacist-led interventions on the mean number of DRPs and the mean QoL score difference per patient DRPs in CKD patients.

## Methods

### Study design

A prospective open-labeled randomized controlled trial was conducted in a single center. Its primary objective is to assess the impact of clinical pharmacist-led interventions, proposed by clinical pharmacist on addressing DRPs among CKD patients to enhance the therapeutic outcome and overall improvement in patient’s QoL.

### Study setting

This trial was performed from April 2023 to July 2023 at North West General Hospital & Research Centre (NWGH& & RC), Peshawar, Pakistan. It is a private hospital offering comprehensive pharmacy services with over 30 full-time pharmacists. However, only the principal investigator, who is a pharmacist at NWGH & RC, was involved in data collection and identification of DRPs.

### Ethics approval

The study received approval from the Institutional Review Board of NWGH & RC Peshawar, Pakistan as well as from the ethics committee of Abdul Wali Khan University Mardan, Pakistan. The trial has been registered with the Australia New Zealand Clinical Trial Registry under trial ID: ACTRN12623000370606, registered on 13/04/2023. As the study involved human participants, all procedures were conducted in adherence to the ethical standards set by the Institutional Review Board/Ethics Committee and in alignment with the guidelines of the 1964 Helsinki Declaration.

Written informed consent was obtained from patients after explaining the objective of this study. Patients can withdraw from the study according to their willingness at any point. In order to ensure patients’ privacy and for future reference, all data was de-identified and each patient was assigned an identification number for follow-up purposes. To minimize any bias the data was kept highly confidential.

### Study population

Adult patients of 18 years and above, of both genders, who were either currently diagnosed with or previously diagnosed with all stages of renal disease and were receiving treatment care at the nephrology unit of NWGH & RC, Peshawar, Pakistan were eligible. Patients without renal disease, pregnant and breastfeeding, and declined to participate were excluded.

### Sample size

A randomized controlled trial conducted by Lenander et al [24] reported that patients at baseline had average of 1.73 ± 0.63 and at endpoint 1.31 ± 0.57 DRPs per patient. Considering 0.05 significance level (α), a power of 95% by using G * power software latest version 3.1.9.7 [25], the sample size we got from calculation was n = 90, having n = 45 in each group. However, keeping in view the 10% dropout rate, a total of n = 100 sample was decided having n = 50 in each group.

### Study procedure

The trial comprised of two phases

### Phase-1: Patient recruitment and screening

A total of N = 150 patients were approached to be assessed for eligibility criteria and consent to participate in this study. Out of these N = 150 patients, N = 30 patients did not meet the inclusion criteria and N = 20 patients refused to participate in this study. All those patients who met the specified set criteria were recruited in this study were N = 100. Consent was taken from the patients who were willing to participate in this study by the principal investigator. The baseline assessment of the hospitalized patients was done, like demographic characteristics, recording of all the medication prescribed to them, laboratory parameters by interviewing patients and also from Hospital Information System (HIS) having electronic health record of patients, assessment of their QoL by using FANLTC questionnaire was recorded.

### Phase-2: Randomization and interventions

To ensure adequate concealment of allocation of the patients to either the control group or clinical pharmacist-led intervention group, the patients handpick an opaque sealed envelope from the basket indicating allocation to either the control or intervention group with 1:1 randomization having N = 50 in each group. The envelope bore a serial number. Upon the patient’s consent to participate in the study, the principal investigator opened the sealed envelope selected by the patient and subsequently assigned them to their designated group according to the group name specified within the envelope. For the patients in intervention group, the DRPs were identified at baseline by a clinical pharmacist. For the identification of DRPs, the PCNE version 9.1, kidney-related clinical practice guidelines, and British National Formulary [BNF] were used as a reference guide for renal dose adjustment of drugs. Furthermore, for the assessment and identification of potential drug-drug interactions and the severity of these interactions Lexicomp was used.

The patients in the control group (N = 50) had usual medication and usual care as per their routine without any involvement of pharmacist interventions for DRPs. The patients in the pharmacist-led intervention group (N = 50) were given a counseling session by the pharmacist with a duration ranging from 15 to 20 minutes without any involvement of clinicians regarding their disease knowledge, medication reconciliation, the need for dose adjustment, and the possible impact of drug-drug interactions on disease progression and their QoL. The recommended interventions for DRPs identified were communicated to the physician/nephrologist upon identification and documented as per PCNE version 9.1 recommended reported guidelines. At the endpoint of the study, i.e., 12 weeks, the patients in both the control group and pharmacist-led intervention group were requested to fill out the QoL assessment questionnaire (FANLTC) to observe any improvement between the baseline and endpoint in their QoL score, keeping in view the interventions recommended for their identified DRPs. For those patients who were discharged from the hospital, their contact details were taken, and were instructed to revisit the hospital after 12 weeks for their assessment related to QoL. This study is only measuring the difference inside one group, i.e., clinical pharmacist-led intervention group for clinical outcome, DRPs, Proposed interventions. However, the QoL score was compared for both control group and intervention group at baseline and endpoint of the study.

### Measurement tools/questionnaires

#### Baseline demographic form.

At baseline demographics characteristics like age, gender, socio-economic status, education status, comorbid conditions, duration of patient lived with diagnosis of CKD, family history, past medical history other than CKD, surgical history and laboratory parameters like serum creatinine, eGFR, complete blood count (CBC), Blood Urea Nitrogen (BUN), uric acid and serum electrolytes, i.e., Potassium, sodium and calcium, and other necessary laboratory findings were obtained from all included patients were collected by the principal investigator. Stages of CKD were categorized based on the Cockcroft-Gault Equation.

#### Dose adjustment and drug interactions.

A data collection form was designed to collect all the medication prescribed to the patients during hospitalization. DRPs and levels of pharmacy intervention were classified, based on the PCNE Scale Version 9.1 [1]. The PCNE classification system for DRPs is a validated and well-established system utilized in diverse healthcare settings. It uses five dimensions to categorize and evaluate DRPs namely “problems (P), causes (C), planned interventions (I), intervention acceptance (A), and status of the DRP (O)” [26].

British National Formulary was used to identify drugs requiring renal dose adjustments, while drug interactions were assessed by using Lexicomp®, which classified them based on interaction severity, and reliability rating [27].

#### Clinical outcomes.

Clinical outcomes including serum creatinine, eGFR, CBC, BUN, and serum electrolytes, i.e., Potassium, sodium, and calcium along with other necessary laboratory findings, were obtained from the medical records of the patients at baseline and endpoint.

#### QoL assessment by Functional Assessment of Non-Life-Threatening Conditions (FANLTC).

The QoL of the patients was assessed using a validated questionnaire using FANLTC [28]. The questionnaire was administered at baseline and endpoint. The FANLTC scale consists of four subscales namely “physical, social/family, emotional, and functional well-being”, respectively, which have a total of 26 questions/items. Each subscale consists of items with different responses ranging from “not at all” which scored 0 to “very much” which scored 4. To determine the scores for positively stated items, a score of 4, 3, 2, 1, and 0 was utilized, whereas the negatively stated items were reverse scored. To calculate the subscale score, the individual scores for all items within each subscale were summed, then multiplied by the number of items in that subscale, and finally divided by the number of items answered. The final QoL score representing the overall assessment using FANLTC was determined by adding up the score of all four subscales, resulting in a score ranging from 0 to 104. A higher FANLTC score indicated a better QoL.

### Statistics analysis

Analysis of the collected data was performed by using IBM “Statistical Package for the Social Sciences” (SPSS) 22^®^ version [29]. Variables such as categorical and continuous variables were presented in the form of frequencies and percentages, mean ± standard deviation (SD). For baseline and endpoint comparison a paired-t test (2-tailed) was performed keeping the significance level less than 0.05. The effect Size within the groups was calculated by using Cohen’s *d* and confidence interval of 95%.

## Results

**Fig 1:**
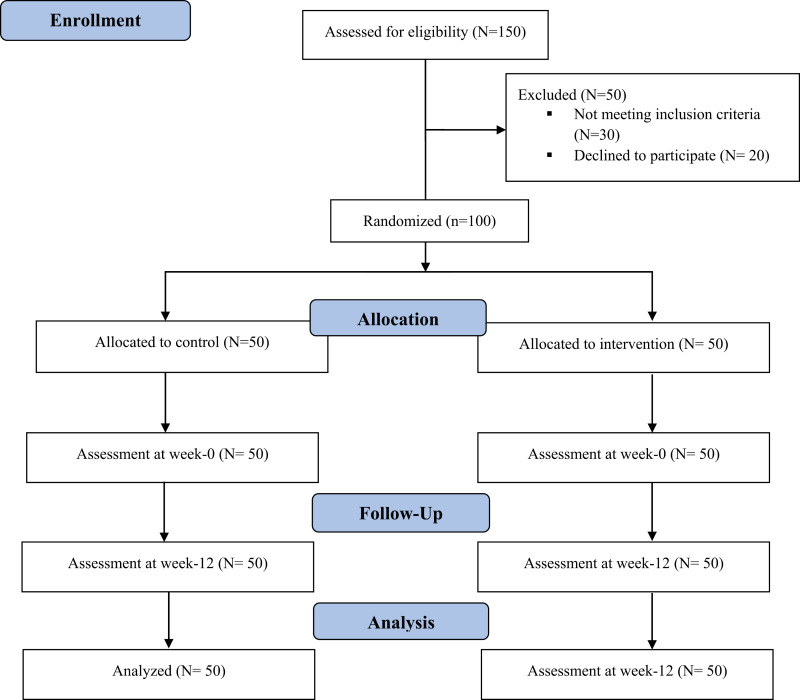
Study flow diagram.

The study included a total of 100 patients who met the specified criteria. These patients were divided into two groups, i.e., control and intervention; each consisting of N = 50 patients. The study flow is presented in Fig 1. Both groups have an equal distribution of males and females. The majority of patients in both groups were aged 60 years and above. In term of education, nearly half of the patients in both groups had a primary level of education, and the majority of the patients in both groups were either jobless, housewives, or students. In the control group, the duration of patient lived with diagnosis of CKD was 56.40 ±  67.84 months; while in the intervention group, it was 57.62 ± 63.08 months [details shown in Table 1].

**Table 1:** Demographic characteristics of participants (n = 100, having n = 50 in each).

	Total patients (n = 100)		Control group (n = 50)		Intervention group (n = 50)	
	n	%	n	%	n	%
**Gender**						
Male	50	50.0	25	50.0	25	50.0
Female	50	50.0	25	50.0	25	50.0
**Age in years**						
< 30 years	15	15.00	8	16.0	7	14.0
30–39 years	15	15.00	9	18.0	6	12.0
40–49 years	11	11.00	4	8.0	7	14.0
50–59 years	24	24.00	11	22.0	13	26.0
60 years and above	35	35.00	18	36.0	17	34.0
**Smoking status**						
No	94	94.0	49	52.1	45	47.9
Yes	6	6.0	1	16.7	5	83.3
**Education level**						
Primary	69	69.0	34	49.3	35	50.7
Secondary	16	16.0	8	50.0	8	50.0
Higher Secondary	9	9.0	5	55.6	4	44.4
Bachelor’s	4	4.0	1	25.0	3	75.0
Postgraduate	2	2.0	2	100	0	0
**Job-status**						
Jobless/Housewife/student	78	78.0	39	50.0	39	50.0
Government job	5	5.0	3	60.0	2	40.0
Private job	7	7.0	1	14.3	6	85.7
Businessman	10	10.0	7	70.0	3	30.0
**Marital status**						
Unmarried	9	9.0	6	66.7	3	33.3
Married	89	89.0	42	47.2	47	52.8
Widow/divorced	2	2.0	2	100.0	0	0
**Number of children’s**						
0	13	13.0	7	53.8	6	46.2
1–2	11	11.0	5	45.5	6	54.5
3–4	27	27.0	13	48.1	14	51.9
5–6	17	17.0	10	58.8	7	41.2
7–8	21	21.0	10	47.6	11	52.4
9–10	11	11.0	5	45.5	6	54.5
**Treatment expenses beard**						
Family	51	51.0	28	54.9	23	45.1
Government	49	49.0	22	44.9	27	55.1
**Duration of patient lived with diagnosis of CKD in months** (Mean ± SD)	56.97 ± 65.33		56.40 ± 67.84		57.62 ± 63.08	
**Frequency of dialysis**						
Not on dialysis	60	60.0	28	46.7	32	53.3
Once a week	2	2.0	1	50.0	1	50.0
Twice weekly	33	33.0	17	51.5	16	48.5
Three times a week	5	5.0	4	80.0	1	20.0
**Stages of CKD**						
Stage 2	4	4.0	1	25.0	3	75.0
Stage 3a	2	2.0	1	50.0	1	50.0
Stage 3b	4	4.0	3	75.0	1	25.0
stage 4	10	10.0	2	20.0	8	80.0
Stage 5	80	80.0	43	53.8	37	46.3

The clinical outcomes of patients at baseline and endpoint for intervention group are presented in Table 2, the findings revealed that there was a statistically significant difference in the following laboratory parameters between the baseline and endpoint, i.e., serum creatinine, urea, potassium, and bicarbonate [as shown in Table 2].

**Table 2:** Clinical outcomes of patients at baseline and endpoint for intervention group only (n = 50).

Variables	Baseline (mean ± SD)	Endpoint (mean ± SD)	p-value
GFR (mL/Min/1.73m^2)	14.74 ± 18.21	18.69 ± 18.49	0.100
Serum Creatinine (mg/dL)	7.39 ± 5.24	5.14 ± 2.78	< 0.001*
Urea (mg/dL)	154.63 ± 90.48	107.72 ± 50.55	0.002*
Hemoglobin (g/dL)	9.46 ± 2.05	9.57 ± 1.57	0.662
WBC (x10^9/L)	10.89 ± 3.84	11.18 ± 5.09	0.643
Platelets (x10^9/L)	289.49 ± 128.86	276.84 ± 137.82	0.413
Sodium (mmol/L)	137.29 ± 5.54	137.31 ± 15.30	0.993
Potassium (mmol/L)	4.81 ± 1.04	4.29 ± 0.84	0.001*
Chloride (mmol/L)	103.03 ± 6.92	103.23 ± 6.17	0.842
Bicarbonate (mmol/L)	20.10 ± 4.35	22.51 ± 4.34	0.009*

Paired t-test was performed; difference is at baseline and at the end of the study; * p-value < 0.05 statistically significant

The incidence of DRPs before and after intervention by cause in the intervention group are listed in Table 3. Overall, there were 230 DRPs identified before the intervention, which decreased to 123 DRPs afterward, indicating a substantial reduction of 46.52%. Under the category of Drug Selection (C1), several subcategories experienced reductions: Inappropriate drug selection according to guidelines/formulary decreased from 73 to 35, representing a reduction of 52.05%; inappropriate combinations of drugs or with herbal medications or dietary supplements decreased from 50 to 20, a reduction of 60.00%; and situations where too many different drugs or active ingredients were prescribed for an indication decreased from 48 to 35, representing a reduction of 27.08% [as shown in Table 3]. Furthermore, in the category of Dose Selection (C3), reductions were seen in instances of both too low dose (from 7 to 3, a reduction of 57.14%) and too high dose (from 19 to 8, a reduction of 57.89%). Lastly, under the category of Others (C9), which encompasses miscellaneous causes, a reduction was observed in other causes (C9.2) from 15 to 6, representing a reduction of 60.00% [details shown in Table 3].

**Table 3:** Incidence of DRPs before and after intervention by cause in intervention group.

Code	Causes (C)	Number of causes		
		Baseline	Endpoint	% reduction
**C**	**Overall DRPs**	**230**	**123**	**46.52**
**C1**	**Drug selection**			
C1.1	*Inappropriate drug according to guidelines/formulary*	73	35	52.05
C1.2	*No indication for drug*	4	3	25.00
C1.3	*Inappropriate combination of drugs, or drugs and herbal medications, or drugs and dietary supplements*	50	20	60.00
C1.4	*Inappropriate duplication of therapeutic group or active* *ingredient*	14	13	7.14
C1.6	*Too many different drugs/active ingredients prescribed for* *indication*	48	35	27.08
**C3**	**Dose selection**			
C3.1	*Too low dose*	7	3	57.14
C3.2	*Too high dose*	19	8	57.89
**C9**	**Others**			
C9.2	*Other causes*	15	6	60.00

The clinical pharmacist provided interventions in order to resolve the DRPs. Interventions recommended were mostly at the prescriber level and drug level. At the prescriber level (I.1): interventions proposed to prescribers (I1.3) accounted for 46.52%, while interventions discussed with prescribers (I1.4) constituted 10.00%. Regarding interventions at the drug level (I.3): dosage changes (I3.2) at 8.26%, Changed instructions for use (I3.4) at 14.35%, drug paused or stopped (I3.5) at 19.13%, and drug started (I3.6) at 1.74%. Acceptance and implementation of Pharmacist Interventions suggested varied: 37.40% of interventions were accepted and fully implemented, 31.30% were accepted and partially implemented, and another 31.30% were accepted but not implemented [details are shown in Table 4].

**Table 4:** Interventions for DRPs resolution provided by clinical pharmacist in the intervention group.

Intervention	Code	Classification	n	%
**I.1 At prescriber level**	I1.3	Intervention proposed to prescriber	107	46.52
	I1.4	Intervention discussed with prescriber	23	10.00
**I.3 At drug level**	I3.2	Dosage changed	19	8.26
	I3.4	Changed instructions for use	33	14.35
	I3.5	Drug paused or stopped	44	19.13
	I3.6	Drug started	4	1.74
	**Total**		**230**	**100%**
**Status of PI**	**Code**	**Classification**	**n**	**%**
**A Acceptance of the intervention by prescriber**	A1.1	Intervention accepted and fully implemented	86	37.40
	A1.2	Intervention accepted, partially implemented	72	31.30
	A1.3	Intervention accepted but not implemented	72	31.30
	**Total**		**230**	**100**

The results showed a statistically significant pharmacist-led intervention effect on FANLTC in all domains including physical wellbeing, social/family wellbeing, emotional wellbeing, and functional wellbeing; the effect size: Cohen’s *d* (95% CI) is shown in Table 5 in the intervention group. Similarly, there was a statistically significant pharmacist-led intervention effect on overall FALTC (QoL score) with effect size: *d, −*1.266, 95% confidence interval [CI], ( *−*1.635; *−*0.889); p-value < 0.001. There was a 27.80% increase in the FANLTC score, which increased (mean ± SD) from 58.64 ± 9.10 at the baseline to 74.48 ± 10.11 at the endpoint; thus, indicating a remarkable improvement in the QoL score [as shown in Table 5].

**Table 5:** Comparison of QoL score for both groups at baseline and endpoint.

Domains	Control group				Intervention group			
	Baseline (mean ± SD) (n = 50)	Endpoint (mean ± SD) (n = 50)	p-value	Effect size: Cohen’s d (95% CI)	Baseline (mean ± SD) (n = 50)	Endpoint (mean ± SD) (n = 50)	p-value	Effect Size: Cohen’s d (95% CI)
Physical wellbeing	12.16 ± 3.44	15.44 ± 3.00	< 0.001*	−0.670 (−0.974; −0.360)	11.16 ± 3.97	18.78 ± 3.01	< 0.001*	−1.858 (−2.315; −1.395)
Social/Family wellbeing	20.86 ± 4.17	21.80 ± 2.32	0.101	−0.236 (−0.516; 0.046)	20.04 ± 3.29	22.26 ± 1.87	< 0.001*	−0.547 (−0.842; −0.247)
Emotional wellbeing	10.04 ± 4.23	12.68 ± 2.88	< 0.001*	−0.575 (−0.872; −0.273)	11.64 ± 2.66	15.20 ± 3.18	< 0.001*	−0.943 (−1.274; −0.606)
Functional wellbeing	14.98 ± 6.57	16.08 ± 6.12	0.148	−0.208 (−0.487; 0.074)	12.76 ± 3.83	18.24 ± 4.97	< 0.001*	−0.937 (−1.267; −0.600)
Overall FANLTC score	61.24 ± 10.18	66.00 ± 9.74	0.002*	−0.475 (−0.766; −0.181)	58.64 ± 9.10	74.48 ±10.11	< 0.001*	−1.266 (−1.635; −0.889)

Paired t-test was performed; * p-value <  0.05 statistically significant

## Discussion

CKD patients due to compromised renal function tend to be at a higher risk for DRPs, of which medication dosing errors are on top [30,31]. Patients who are hospitalized with CKD are at a higher risk of experiencing drug duplication, interactions, and adverse events. These risks can lead to prolonged hospitalization, increased expenses, and a reduced QoL. Patients with CKD experience other complications including several comorbidities [32–34], including diabetes mellitus [30,33], cardiovascular disease [35,36], and hypertension that further exacerbate the consequences of impaired kidney function [37,38]. Thereby, polypharmacy is inevitable and highly prevalent among these patients. The use of multiple medications for managing comorbidities further exacerbates the progression of CKD [33]. Pakistan being a developing country witnessed a big challenge in the form of inappropriate dose adjustment of drugs among the CKD patients due to overburdened and limited number of nephrologists [39]. In both developing countries as well as developed countries, around 25%-77% of drugs are adjusted inappropriately [40,41]. For better clinical outcomes it is crucial to identify, prevent, and resolve DRPs among CKD patients [15]. To optimize medication management in CKD patients, effective collaboration among clinicians, clinical pharmacists, and other healthcare professionals is essential. This interdisciplinary approach facilitates proactive and comprehensive care for CKD patients regarding their medications. Therefore, this study aimed to assess the impact of clinical pharmacist-led interventions on identifying and resolution of DRPs and the QoL of CKD patients during their hospital stay.

In our study, the pharmacist identified a total of n = 230 DRPs in the intervention group and majority of the DRPs were attributed to “inappropriate drug selection according to guidelines/formulary”; “inappropriate combinations of drugs or with herbal medications or dietary supplements”; and situations where “too many different drugs or active ingredients were prescribed”. Furthermore, in the category of Dose Selection too low dose and too high dose were also attributing to DRPs. However, previous studies with similar objectives identified a significant number of DRPs, with inappropriate drug selection/dosing and drug interactions being the primary sources of concern [42]; Similarly, another study reported that the DRPs observed in their research were primarily related to drug interactions, followed by frequency errors and indications without corresponding drugs [43]. Polypharmacy can be considered an important factor for causing DRPs and in our study majority of the patients were prescribed with higher number of drugs. Our study findings are aligned with study findings of another study [44]. In our study due to pharmacist intervention there was 46.52% reduction to total DRPs, which are align with other study having 65.53% reduction in DRPs [16]. It is pertinent to mention that in our study pharmacist identified DRPs in considerable number of patients which is consistent with other studies wherein clinical pharmacist promoted the safe effective and economic use of drugs [18–20]. However, identification and prevention of DRPs also depends on the competencies, skills and knowledge of clinical pharmacist, lack of required competencies may compromise outcomes.

Our study findings revealed that the interventions proposed by the clinical pharmacist were not implemented in true letter and spirit. Our study findings revealed that only 37.40% of the proposed interventions were fully implemented by the prescribers while 31.30% were partially implemented and 31.30% of the proposed interventions were not implemented at all by the prescribers. Our proposed interventions were significantly lower as compared to another recent study where 95.8% of the proposed interventions were accepted and implemented by the prescriber [45]. Additionally, another study documented 100% acceptance and implementation of the proposed intervention by the prescriber [16]. Moreover, another study revealed that 11.06% of interventions were accepted and completely implemented by the prescribers, while 54.46% of the interventions were accepted and partially implemented and 34.48% of the interventions were accepted but not implemented [16]. The possible reason for the low acceptance and complete implementation of the proposed intervention in our study could be attributed to the prevailing perception that pharmacists in Pakistan are only limited to dispensing and inventory management of medicines. A study conducted in South Korea strengthen our stance for low acceptance of interventions, where pharmacists were primarily restricted to tasks like dispensing medications and providing drug counseling [46].

As far as the QoL of CKD patients is concerned, our study findings revealed a significant improvement in the QoL of patients between baseline and endpoint in the pharmacist-led intervention group, while the QoL score in control group having usual care improved from 61.24 ± 10.18 at baseline to 66.00 ± 9.74 at the endpoint of study. Our findings revealed that due to the full and partial implementation of proposed interventions in the pharmacist-led intervention group, the mean ± SD for the QoL score increased from 58.64 ± 9.10 at the baseline to 74.48 ± 10.11 at the endpoint. Our study findings are aligned with other studies, in which the QoL score showed a statistically significant improvement in QoL score at the endpoint following pharmacist interventions [46,47]. The improvement in QoL of patients in the intervention group is proposed to be due to the resolution of DRPs by the prescriber. As stated above a substantial percentage of proposed interventions were either partially implemented or not implemented as proposed by clinical pharmacist, if these proposed interventions had been implemented in 100%, the QoL of the patients would have certainly improved to its maximum.

### Strength and limitations

This study has a notable strength as it represents the first of its kind conducted in Pakistan, aiming to evaluate the impact of pharmacist-led interventions in identifying DRPs, proposing interventions, and assessing the QoL of CKD patients. The study also examines the changes in QoL, and highlights the significance of clinical pharmacists in delivering clinical services. This study has some limitations that need to be considered. Firstly, it was conducted at a single center, specifically the nephrology unit of NWGH & RC in Peshawar, Pakistan; although a significant number of patients were recruited from the specified center; it would be beneficial to conduct a multicenter study by recruiting a large sample of patients to analyze the impact of pharmacist-led interventions among the CKD patients.

## Conclusions

In this study we observed a significant improvement in the QoL for patients with CKD following clinical pharmacist-led interventions when the proposed interventions were implemented successfully; however, a considerable number of proposed interventions were not accepted and implemented. Hence, to rationalize the prescriptions and achieve better therapeutic outcomes as well as improved patients’ QoL, it is strongly recommended to adopt a collaborative and multidisciplinary approach, and involve pharmacists in clinical services. This is particularly important because pharmacists in hospital pharmacies are often confined to tasks such as drug dispensing and counseling only.

## Supporting information

S1 Text Inclusivity in global research. (DOCX)

S2 Text CONSORT-2010-Checklist. (DOCX)

S3 Text Protocol for RCT. (DOCX)

S1 Data Supplementary data. (SAV)
