# Supplementary material for: Impact of pharmacist-led intervention for reducing drug-related problems and improving quality of life among chronic kidney disease patients: A randomized controlled trial
Source: PLoS One. 2025 Feb 3;20(2):e0317734. doi: 10.1371/journal.pone.0317734 (PMC11790119; doi:10.1371/journal.pone.0317734)
Supplement: S3 Text — (DOCX) [file pone.0317734.s003.docx]

**Impact of pharmacist-led intervention for reducing drug related problems and improving quality of life among chronic kidney disease patients. A randomized controlled trial**

**Aim of study**

**Primary aim**

Impact of pharmacist led interventions for drug related problems (including dose related, drug interactions, side effect or if any) in chronic kidney disease patients based on the Pharmaceutical care network Europe association (PCNE) scale version 9.1 [1].

**Secondary aim**

Assessment for quality of life of chronic kidney disease patients by using the Functional Assessment of Non-Life Threatening Conditions (FANLTC) questionnaire [2] to compare the impact of pharmacist counseling and adjustment of drug related problems at baseline and endpoint.

**Method
Study design**

This is a multi-centered, open-label, parallel group, prospective randomized control trial. In this study Impact of pharmacist led interventions for drug related problems among chronic kidney disease patients were investigated to address the drug related problems including dose adjustment, drug interactions in order to improve therapeutic outcome and overall improvement in patient’s quality of life. The purpose of the study was explained, and patients consent was obtained before enrolling by the principle investigator. The study flow is presented in Figure 1 below.

**Study setting**

This trial would be conducted from April 2023 to July 2023 at North West General Hospital & Research Center, Peshawar, Pakistan.

**Ethics and dissemination**

The study protocol was approved by the institutional review board of North West General Hospital & Research center Peshawar, Pakistan and Ethics Committee of Abdul Wali Khan University Mardan, Pakistan. The trial is registered under Australia New Zealand Clinical Trial Registry Trial ID: ACTRN12623000370606, on 13/04/2023. As the study involved human participants, therefore all procedures were performed in accordance with the ethical standards of the institutional
research committee and also to be compliant with the 1964 Helsinki declaration. After explaining the purpose of study to all the patients, informed written consent was obtained from all patients. The patients were allowed to withdraw from the study according to their willingness at any point of the study. For track record of patient and future reference, all data was de-identified and only identification number were allocated to each patient for follow up when required. To minimize any bias the data was kept highly confidential.

**Study population**

Eligibility criteria for the patients recruitment includes adult patients of 18 years and above, both genders who currently had or previously diagnosed with all stages of renal disease and hospitalized in the nephrology unit of North West General Hospital & Research Center, Peshawar, Pakistan. Patients not having renal disease, those who are pregnant and breastfeeding and not willing to participate.

**Study procedure**

This trial has 2 phases,

**Phase 1: (recruitment and screening of patients)**

All those patients meeting the inclusion criteria were recruited to participate in this study. The objective of the study was explained to them, after their willingness to participate, a written informed consent was obtained from each individual patient. The baseline assessment of the patients was done, like demographic characteristics, recording of all the medication prescribed to them, assessment of their quality of life was recorded.

**Assessment for eligibility**

**Excluded**

Not meeting inclusion criteria

Enrollment

**Randomized**

Allocated to control group

Allocated to intervention group

Allocation

0 weeks

Follow up

0 weeks

12 weeks

12 weeks

Analysis

Analysis

Analysis

*Figure 1: Flow chart of Randomized control trial for pharmacist led intervention model*

**Phase 2: (Randomization and interventions)**

To ensure adequate concealment of allocation, the patients were requested to handpick an envelope from the basket indicating allocation to groups.

The patients were randomized 1:1 into two groups, either control group or intervention group. For the patients in intervention group, the drug related problems were identified at baseline by clinical pharmacist. The PCNE version 9.1 and kidney-related clinical practice guidelines were used as guides regarding dose adjustment of drugs requiring renal adjustment and drug interactions based on the PCNE Scale version 9.1, British National Formulary [BNF]. Furthermore, for assessment and identification of potential drug –drug interactions and severity of these interactions Lexicomp was used. The proposed interventions were communicated to the physician/nephrologist upon identification and documented as per PCNE version 9.1 recommended reported guidelines. The patients in the control group had usual medication and usual care as per their routine without any involvement of pharmacist interventions for DRPs. The patients in the pharmacist-led intervention group were given a counseling session by the pharmacist with a duration ranging from 15 to 20 minutes without any involvement of clinicians regarding their disease knowledge, medication reconciliation, the need for dose adjustment, and the possible impact of drug-drug interactions on disease progression and their Quality of life.

At endpoint of study the patients in both groups control and intervention group were requested to fill the quality-of-life assessment questionnaire to record the difference and improvement if any between the baseline and endpoint.

**Measurement tools**

1. ***Baseline demographic form***

At baseline demographics characteristics like age, gender, socio-economic status, education status, comorbid conditions, duration of CKD, family history, past medical history other than CKD, surgical history and laboratory parameters like serum creatinine, estimated glomerular filtration rate, complete blood count, Blood Urea Nitrogen, uric acid and serum electrolytes i.e., Potassium, sodium and calcium, and other necessary laboratory findings were obtained from all included patients. Stages of CKD were categorized based on Cockcroft Gault Equation.

1. ***Dose adjustment and drug interactions***

A data collection form was designed to collect all the medication prescribed to the patients. Drug related problems and levels of pharmacy intervention were classified, based on the PCNE Scale Version 9.1 [1]. The PCNE drug related problems (DRPs) classification system is a validated DRP classification used in a variety of settings, and it includes five domains: problems (P), causes (C), planned interventions (I), intervention acceptance (A), and status of the DRP (O) [3]. British National Formulary was used to identify drugs requiring renal dose adjustments, while drug interactions were assessed by using Lexicomp®, which classified them based on interaction severity, and reliability rating [4].

1. **Clinical outcomes**

Clinical outcomes such as blood glucose level, serum creatinine, estimated glomerular filtration rate, complete blood count, Blood Urea Nitrogen, uric acid and serum electrolytes i.e., Potassium, sodium and calcium, and other necessary laboratory findings were obtained from the computer and medical records of the patients. These lab parameters were measured at the baseline and at end point of the study. The changes in the value of the clinical parameters were analyzed to find if there is any significant difference after the intervention.

1. ***Quality of life assessment by Functional Assessment of Non-Life Threatening Conditions (FANLTC)***

The QOL of the study participants was assessed using a validated questionnaire using FANLTC both at baseline and end point [2]. The FANLTC questionnaire comprised of 4 subscales i.e., “physical well-being, social/family well-being, emotional well-being, and functional well-being “, which have a total of 26 questions/items. Each subscale consist a items with different responses ranging from “not at all” which was scored 0 to “very much” was scored 4. The scoring of positively stated items is 4, 3, 2, 1, and 0, while the negatively stated items was reverse scored. The calculate the subscale score, the score of all items in individual subscale were then summed, then multiply by the number of items in every subscale and divided by the number of answered items. The final and overall score of FANLTC questionnaire representing QOL was calculated by totaling the score of all the 4 subscales, which ranges from 0 to 104. The higher FANLTC score reveals better quality of life.

**Sample size**

From previous study, patients had a mean of 1.73 ± 0.63 drug related problems per patient at baseline [5]. Assuming α=0.05, while estimating having a 90% power to detect a 23% reduction in the number of DRPs between the intervention and control group with 10% dropout rate [6], the total sample size was n=100, having control group =50 and intervention group=50.

**Statistics analysis**

Statistical analysis was performed by using SPSS 22, categorical variables will be expressed as frequency and percentages; mean ± standard deviation for continuous variables. For comparison of baseline and endpoint parameters like laboratory parameters and quality of life score, a pair-t test was performed while the significance level was set at p < 0.05.

**Discussion**

CKD patients due to comprised renal function tend to be at a higher risk for drug-related problems, of which medication dosing errors are on top [7, 8]. Hospitalized patients with CKD have a high risk of drug duplication, interactions, and adverse events, which could result in extended hospital stays, higher costs, and low quality of life. Patients with CKD experience other complications including several comorbidities [9-11], including diabetes mellitus [10, 12], cardiovascular disease (CVD) [13, 14], and hypertension that further exacerbate the consequences of impaired kidney function [15, 16]; therefore, polypharmacy is inevitable and highly prevalent among these patients. The use of multiple medications for managing comorbidities further exacerbates the progression of CKD [10]. The identification, prevention and resolution of DRPs problems are very important for CKD patients, which in turn will lead to better clinical outcomes [17].

Pakistan being a developing country witness a big challenge in form of inappropriate dose adjustment of drugs among the CKD patients due to overburdened and limited number of nephrologist [18]. In both developing countries as well as developed countries, round 25%-77% of drugs are adjusted inappropriately [19, 20]. Furthermore, CKD patient suffer from different comorbidities for which polypharmacy is inevitable, thus the risk of pDDIs increases with the number of drugs prescribed [21]. Pharmacist involvement and recommendation of intervention regarding the pharmacotherapy recommendations, dosage adjustments of prescribed medication according to kidney function and monitoring of laboratory parameters [22, 23] and literature supported a significantly reduction in DRPS resulting from pharmacist interventions [6]. Lack of pharmacists in clinical settings, absence of a national dosing formulary, and computerized dose adjustment programs in Pakistan lead to higher medication dosing error [20]. Medication optimization in CKD patients requires a proactive interdisciplinary collaboration between clinicians, clinical pharmacists, and other healthcare professionals.

1. *Pharmaceutical Care Network Europe (PCNE). The PCNE classification V9.00.* . Available from: <https://www.pcne.org/upload/files/417_PCNE_classification_V9-1_final.pdf>.

2. Rehman, I.U., et al., *The association between CKD-associated pruritus and quality of life in patients undergoing hemodialysis in Pakistan: A STROBE complaint cross-sectional study.* Medicine, 2019. **98**(36): p. e16812-e16812.

3. Liu, P., et al., *Identification and solution of drug-related problems in the neurology unit of a tertiary hospital in China.* BMC Pharmacology and Toxicology, 2021. **22**(1): p. 1-9.

4. Nusair, M.B., et al., *The prevalence and severity of potential drug-drug interactions among adult polypharmacy patients at outpatient clinics in Jordan.* Saudi Pharmaceutical Journal, 2020. **28**(2): p. 155-160.

5. Lenander, C., et al., *Effects of a pharmacist-led structured medication review in primary care on drug-related problems and hospital admission rates: a randomized controlled trial.* Scandinavian Journal of Primary Health Care, 2014. **32**(4): p. 180-186.

6. Song, Y.-K., et al., *Effectiveness of Clinical Pharmacist Service on Drug-Related Problems and Patient Outcomes for Hospitalized Patients with Chronic Kidney Disease: A Randomized Controlled Trial.* Journal of clinical medicine, 2021. **10**(8): p. 1788.

7. Saad, R., S. Hallit, and B. Chahine, *Evaluation of renal drug dosing adjustment in chronic kidney disease patients at two university hospitals in Lebanon.* Pharmacy Practice (Granada), 2019. **17**(1).

8. Saleem, A. and I. Masood, *Pattern and predictors of medication dosing errors in chronic kidney disease patients in Pakistan: a single center retrospective analysis.* PLoS One, 2016. **11**(7): p. e0158677.

9. Jhee, J.H., et al., *Intensity of statin therapy and renal outcome in chronic kidney disease: Results from the Korean Cohort Study for Outcome in Patients With Chronic Kidney Disease.* Kidney research and clinical practice, 2020. **39**(1): p. 93.

10. Schmidt, I.M., et al., *Patterns of medication use and the burden of polypharmacy in patients with chronic kidney disease: the German Chronic Kidney Disease study.* Clinical kidney journal, 2019. **12**(5): p. 663-672.

11. Titze, S., et al., *Disease burden and risk profile in referred patients with moderate chronic kidney disease: composition of the German Chronic Kidney Disease (GCKD) cohort.* Nephrology Dialysis Transplantation, 2015. **30**(3): p. 441-451.

12. Li, J., et al., *Prevalence and predictors of polypharmacy prescription among type 2 diabetes patients at a tertiary care department in Ningbo, China: a retrospective database study.* PLoS One, 2019. **14**(7): p. e0220047.

13. Al-Shamsi, S., D. Regmi, and R. Govender, *Chronic kidney disease in patients at high risk of cardiovascular disease in the United Arab Emirates: A population-based study.* PloS one, 2018. **13**(6): p. e0199920.

14. Harężlak, T., et al., *Drug interactions affecting kidney function: Beware of health threats from triple whammy.* Advances in Therapy, 2022: p. 1-8.

15. Shahzadi, A., et al., *The Prevalence of Potential Drug-Drug Interactions in CKD-A Retrospective Observational Study of Cerrahpasa Nephrology Unit.* Medicina, 2022. **58**(2): p. 183.

16. Scheppach, J.B., et al., *Blood pressure pattern and target organ damage in patients with chronic kidney disease.* Hypertension, 2018. **72**(4): p. 929-936.

17. Shouqair, T.M., et al., *Evaluation of Drug-Related Problems in Chronic Kidney Disease Patients.* Cureus, 2022. **14**(4).

18. Anees, M., M. Ibrahim, and M. Nazir, *Comparison of awareness about nephrology and kidney diseases amongst doctors in institutes with and without nephrology departments.* Pakistan Journal of Medical Sciences, 2014. **30**(4): p. 891.

19. Fink, J.C. and G.M. Chertow, *Medication errors in chronic kidney disease: one piece in the patient safety puzzle.* Kidney international, 2009. **76**(11): p. 1123-1125.

20. Hassan, Z., et al., *Assessment of medication dosage adjustment in hospitalized patients with chronic kidney disease.* Cureus, 2021. **13**(2).

21. Avery, A.A., et al., *Investigating the prevalence and causes of prescribing errors in general practice: the PRACtICe study.* 2012.

22. Al Raiisi, F., et al., *Clinical pharmacy practice in the care of Chronic Kidney Disease patients: a systematic review.* International journal of clinical pharmacy, 2019. **41**: p. 630-666.

23. Joy, M.S., et al., *Clinical pharmacists as multidisciplinary health care providers in the management of CKD: a joint opinion by the Nephrology and Ambulatory Care Practice and Research Networks of the American College of Clinical Pharmacy.* American journal of kidney diseases, 2005. **45**(6): p. 1105-1118.
